# Usefulness and safety of midline incision for right-sided hepatectomy: Cohort study

**DOI:** 10.1016/j.amsu.2021.102498

**Published:** 2021-06-13

**Authors:** Daisuke Takei, Shintaro Kuroda, Keiso Matsubara, Hiroaki Mashima, Masakazu Hashimoto, Tsuyoshi Kobayashi, Hideki Ohdan

**Affiliations:** Department of Gastroenterological and Transplant Surgery, Graduate School of Biomedical and Health Science, Hiroshima University, Hiroshima, Japan

**Keywords:** Liver cancer, Hepatectomy, Midline incision, Hepatocellular carcinoma, Post-operative pain

## Abstract

**Background:**

While the adoption rates of laparoscopic hepatectomy are increasing, most patients still undergo open hepatectomy. Open hepatectomies use inverted L-shaped or Mercedes incisions for right-sided liver tumor. To decrease procedural invasiveness, we performed midline incisions in such cases, excluding those of laparoscopic hepatectomy. This retrospective study examined the effects of this change in treatment policy on overall patient surgical outcomes.

**Materials and methods:**

From 2012 to 2018, 374 patients who underwent hepatectomy for right-sided hepatocellular carcinoma were enrolled, and short-term patient outcomes were compared following stratification into the 1st (n = 157) or 2nd (n = 217) Era group based on whether procedures occurred before or after the policy change, respectively.

**Results:**

Short-term outcomes were mostly comparable between the two groups, with significantly increased postoperative aspartate aminotransferase maximum values found in the 2nd Era group relative to the 1st Era group (median: 393 vs. 331, p < 0.05). Pain scores at rest during postoperative day 1 and while moving on postoperative days 1, 2, and 3 were significantly lower in the 2nd Era group than in the 1st Era group (p < 0.05, <0.01, <0.05, <0.01, respectively).

**Conclusions:**

Utilization of midline incisions may provide some benefits in postoperative outcomes for right-sided open hepatectomy cases.

## Highlights

• Utilization of midline incisions may provide better postoperative outcomes in cases of right-sided open hepatectomy.

## 1 Introduction

Minimally invasive surgeries, including laparoscopies, have been widely adopted across many surgical fields [[1], [2], [3], [4], [5], [6]]. However, laparoscopic hepatectomy adoption rates for liver cancer remain low due to concerns regarding potential technical difficulties and the ability to accurately identify resection range. Challenges associated with performing laparoscopic hepatectomy for liver cancers include alterations in approach based on tumor location, cirrhosis-associated bleeding risks, presence of giant tumors, and the potential need to perform anatomical resection or repeat hepatectomy [[7], [8], [9], [10], [11], [12], [13]]. Data from the Japanese national clinical database showed an increase in the number of laparoscopic surgeries performed for liver resections from 1848 (9.9%) in 2011 to 5648 (24.8%) in 2017 [14]. The most common incisions used in open hepatectomy for right-sided liver cancers include the inverted L-shape and Mercedes incisions. Recently, hepatectomies performed through an upper midline incision have been introduced as a less invasive approach for harvesting liver grafts from living donors [[15], [16], [17], [18], [19], [20], [21], [22]]. Since 2015, we adopted the use of an upper midline incision for hepatectomy as a less invasive surgery for patients with liver cancer, including right-sided liver cancers, except in cases of laparoscopic hepatectomy. Difficulties in using upper midline incision in hepatectomy cases for treating liver cancer are dependent on tumor location and considerations of whether anatomical or non-anatomical resections are required. Visibility and accessibility are concerns during hepatectomy, particularly in cases involving posterior segment resections when working in a deep surgical field. Our institution previously performed hepatectomies using the inverted L-shaped incisions except in cases of laparoscopic surgeries before modifying treatment policies in 2015 to implement the use of midline incisions where possible to reduce overall invasiveness. This study aimed to examine changes in postoperative pain and safety associated with liver resection resulting from this change in treatment policy to determine whether switching to less invasive midline incisions would result in more positive patient outcomes.

## 2 Material and methods

### 2.1 Patient selection

**Fig. 1:**
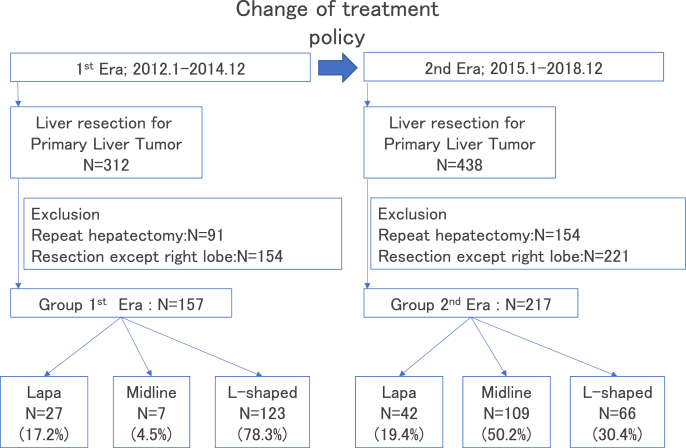
Change in the institutional treatment policy The first-choice surgical strategy is laparoscopic surgery; however, since January 2015, laparotomies were performed utilizing midline incisions where possible.

We retrospectively enrolled 750 patients who underwent hepatectomy for hepatocellular carcinoma (HCC) between January 2012 and December 2018. Of these, we excluded patients who underwent repeat hepatectomy (n = 245) or cases that did not include right lobe excision (n = 375), resulting in a final study population of 374 patients. Additionally, all patients who were eligible for laparoscopic procedures underwent laparoscopic surgery. Patients recruited after the policy change went into effect in January 2015 underwent procedures that mainly utilized upper midline incisions, except for those who underwent laparoscopic surgeries. Patients were stratified based on the timing of the procedure (before or after the policy change). The 1st Era group (L-shaped incision: n = 123; Midline incision: n = 7; Laparoscopic surgery: n = 27) comprised patients who underwent procedures before the institutional policy change and the 2nd Era group (L-shaped incision: n = 66; Midline incision: n = 109; Laparoscopic surgery: n = 42) comprised patients who underwent procedures after the policy change (Fig. 1). This study conforms to the provisions of the Declaration of Helsinki and was approved by the local Institutional Review Board. All patients provided informed consent before undergoing their respective surgical procedures. All surgical procedures were performed by a different group of surgeons practicing at our institution.

### 2.2 Surgical procedure

**Fig. 2:**
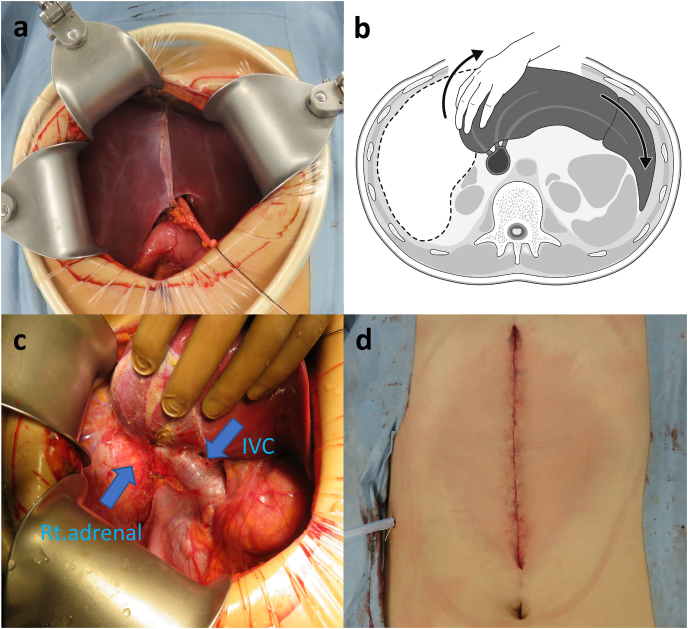
Surgical procedure for hepatectomy utilizing an upper midline incision for right-sided liver cancer (a) The abdominal wall is retracted using the Thompson retractor. (b) The entire liver is rotated from the right to the left side in order to bring the resection range to just below the wound. (c) The right adrenal gland is dissected in order to fully mobilize the right lobe. (d) Postoperative image overview of the wound following right-sided hepatectomy utilizing an upper midline incision.

For patients undergoing hepatectomy using upper midline incisions, the incision was made from the xiphoid process to a point above the umbilicus. A Thompson retractor (Thompson Surgical Instruments Inc., Traverse City, MI, USA) was used to retract the abdominal wall (Fig. 2a), and the left coronary and triangular ligaments were separated to free the left hepatic lobe to fully mobilize the right hepatic lobe. Next, the right coronary and triangular ligaments were divided, and the Thompson retractor was pulled to the right while rotating the liver gradually to the left (Fig. 2b), allowing the right lobe to be separated from the diaphragm and retroperitoneal space. The right side of the inferior vena cava (IVC) was taped to the right adrenal gland, dissected or sutured, and ligated (Fig. 2c) to allow for the mobilization of the right lobe. Liver mobilization was facilitated through incremental movements using thing IVC as the axis.

For conventional inverted L-shaped incision hepatectomy, the upper midline incision was extended transversely from the umbilicus to the ninth intercostal space, while a Kent retractor (Takasago Medical Industry Co., Tokyo, Japan) was used for abdominal wall retraction.

Hepatectomies were performed uniformly, regardless of the incision type. Intermittent inflow occlusions were achieved either by using the Pringle method, wherein the hepatoduodenal ligament is clamped to temporarily block the blood flow in the hepatic artery and portal system or clamping the inferior vena cava, and the parenchyma was dissected using a cavitation ultrasonic aspiration system (CUSA Excel; Integra Life Sciences Co., Plainsboro, NJ, USA) or water jet (ERBE, Inc., Tübingen, Germany). For anatomical resections, a Glissonean pedicle approach at the porta hepatis is used under normal conditions [23]. However, if there were concerns regarding liver hardness or the branching form of the Glisson's capsule, the resected region was identified by staining and was guided using ultrasound [24]. In addition, the hanging maneuver was performed as needed. A drain was placed on the remaining liver segment during the surgical closure and remained in place until postoperative day 3 (Fig. 2d). For both groups, patients were first evaluated for laparoscopic surgery eligibility. Before the treatment policy change, for cases where laparoscopic surgery was determined to be challenging, an L-shaped laparotomy was performed. Following the implementation of the new treatment policy, midline incisions were utilized whenever possible. However, an L-shaped laparotomy transition was unavoidable in some cases due to a poor field of view.

### 2.3 Measurement and assessment of postoperative pain

Pain management and postoperative pain assessments remained consistent for both treatment groups. Specifically, epidural (Epi)–patient-controlled analgesia (PCA) was generally used for patients undergoing open hepatectomy. Intravenous (IV)-PCA was used in patients undergoing anti-coagulation or anti-platelet therapy or those who had thrombocytopenia (platelet count of 80,000 or less or a prothrombin time of 80% or less) to minimize the risk of postoperative epidural hematomas. In addition to narcotics (fentanyl) used in PCA, non-steroidal anti-inflammatory drugs (NSAIDs) or acetaminophen were also administered twice daily at fixed intervals, beginning after postoperative day 2. PCA was terminated on postoperative day 4 or 5, depending on patient-reported pain levels. Postoperative pain assessments using the visual analogue scale (VAS) were performed by anesthesiologists twice daily while patients were at rest or undergoing movement-related activities on postoperative days 1, 2, and 3 [25].

### 2.4 Definitions

Indications for the requirement of hepatectomy were determined based on the tumor and resultant liver dysfunction according to the Child-Pugh classiﬁcation, comprising five variables: encephalopathy, ascites, serum bilirubin, serum albumin and prothrombin activity, and the Liver Damage Grade, measuring indocyanine green retention rates at 15 min (ICGR-15) instead of encephalopathy [26]. Patients with sufficient liver function underwent anatomic resections, while partial resections were performed in patients with insufficient liver function.

A right-sided hepatectomy was defined as a resection of segments 5, 6, 7, and 8 based on the Couinaud classification. In some patients, both right- and left-sided hepatectomies were performed. Major hepatectomy was deﬁned as the resection of three or more segments, while minor hepatectomy was deﬁned as the resection of no more than two segments.

The Clavien-Dindo classification and post-hepatectomy liver failure (PHLF) definitions were used to evaluate surgical complications. If the patient required surgical, endoscopic, or radiological intervention, the complication was assigned a Clavien-Dindo grade of III or more [27]. The PHLF was considered positive when the ICGR-15 and total-bilirubin levels on postoperative day 5 were above the normal limits [28].

### 2.5 Statistical analyses

Continuous variables are presented as medians and ranges. For continuous variables, parametric analyses were performed using Student's *t*-test for normally distributed data, while the Mann–Whitney *U* test was used for non-parametric analyses. Categorical variables were compared using Fisher's exact test.

To account for biases resulting from differences in covariate distribution across patients in the 2nd Midline and 1st L-shaped groups, one-to-one matching was conducted using a propensity score analysis. Variables entered in the propensity model were prothrombin time as the preoperative factor, while tumor size and resection weight were used as operative factors.

All statistical analyses were conducted using JMP Genomics version 14 (SAS Institute Inc., Cary, NC, USA); p-values of <0.05 were considered to be statistically significant. The work has been reported in line with the STROCSS criteria [29]. This study is registered with the Research Registry, and the UIN is researchregistry6476 (https://www.researchregistry.com/register-now#user-researchregistry/registerresearchdetails/6007c7d7dbe65d001cb358ac/).

## 3 Results

### 3.1 Study population and clinical characteristics

Clinical characteristics of the study population are summarized in Supplemental Table 1. We enrolled 287 men (76%) and 87 women (24%) with a median age 70 years (range: 20–91 years), and a median body mass index (BMI) of 23.1 kg/m^2^ (range: 15.5–37.3 kg/m^2^). Of the 374 patients, 348 (93%) were classified as Child-Pugh grade A and 26 (7%) were classified as Child-Pugh grade B.

The median tumor size was 25 mm (range: 8–200 mm), with liver tumor numbers ranging from 1 to 20. A single liver tumor was found in 230 patients (61%), and multiple liver tumors were found in 144 patients (39%). Liver cirrhosis was present in 75 patients (20%). Posterior tumors were present in 190 patients (51%), and the remaining 184 patients (49%) had tumors in other regions. Anatomical resections were performed in 261 patients (69%), and limited resections were performed in 113 patients (31%). Epi-PCA and IV-PCA were used by 193 patients (52%) and 180 patients (48%), respectively.

### 3.2 Era group-stratified clinical characteristics and outcomes

To determine whether changes in treatment policies had effects on postoperative outcomes, we compared the short-term outcomes between patients in the 1st and 2nd Era groups, which included cases that utilized midline incisions, L-shaped incisions, or laparoscopic hepatectomy, using an intention to treat (ITT) analysis.

Comparisons of clinical characteristics across the 1st and 2nd Era groups are shown in Table 1. Most preoperative and operative factors were similar across the groups; however, the 2nd Era group had significantly lower aspartate aminotransferase (AST) and α-fetoprotein (AFP) levels (AST median: 36 vs. 31, p < 0.05 U/L; AFP median: 14 vs. 7 ng/mL, p = 0.01) with a longer prothrombin time (median (%): 84 vs. 90, p < 0.01) than did the 1st Era group.

**Table 1:** Clinical characteristics of patients in the 1st Era and 2nd Era groups.

Variables	Group 1st Era (n = 157)	Group 2nd Era (n = 217)	P-Value
Age^a^	70 (33–91)	71 (20–88)	0.24
Sex; Male/Female	122/35	165/52	0.70
BMI^a^	22.8 (16.5–37.3)	23.2 (15.5–36.9)	0.12
DM; Yes/No	48/68	66/113	0.43
T-Bil (mg/dl)^a^	0.8 (0.3–2.9)	0.8 (0.3–2.7)	0.76
AST (IU/l)^a^	36 (11–296)	31 (12–390)	<0.05
Albumin (mg/dl)^a^	4.1 (2.3–5.1)	4.0 (2.5–7.4)	0.92
Prothrombin time (%)^a^	84 (24–112)	90 (24–145)	<0.01
ICGR-15 (%)^a^	13.8 (2.6–79.1)	13.2 (2.2–79.2)	0.85
Child-Pugh classification; A/B	143/14	205/12	0.20
AFP (ng/ml)^a^	14 (0.5–11234)	7 (0.5–290700)	<0.05
Tumor size (mm)^a^	25 (8–170)	25 (8–200)	0.73
Tumor number; Single/Multiple	97/60	133/84	0.92
Liver cirrhosis; Yes/No	36/121	39/178	0.23
Posterior region; Yes/No	71/86	113/104	0.19
Resected weight (g)^a^	151 (5–2332)	137 (6–2903)	0.79
Type of hepatectomy; Anatomical/Limited	107/50	154/63	0.55
Type of hepatectomy; Major/Minor	16/141	22/195	0.98
Perioperative pain management; Epi-PCA/iv-PCA	80/77	113/103	0.83

The 1st Era group (L-shaped incision: n = 123; Midline incision: n = 7; Laparoscopic surgery: n = 27) comprised patients who underwent procedures prior to the institutional policy change. The 2nd Era group (L-shaped incision: n = 66; Midline incision: n = 109; Laparoscopic surgery: n = 42) comprised patients who underwent procedures after the policy change.

BMI, body mass index; DM, diabetes mellitus; T-Bil, total bilirubin; AST, aspartate aminotransferase; ALT, alanine transaminase; ICGR-15, indocyanine green retention rate at 15 min; AFP, α-fetoprotein; Epi-PCA, epidural-patient-controlled analgesia; iv-PCA, intravenous-patient-controlled analgesia.

a [median, (range)].

We next compared the surgical and postoperative outcomes across the two groups. There were no significant differences with regard to surgical parameters (operative time, blood loss) and most postoperative outcomes (length of hospitalization, complications, and PHLF grade) (Table 2). However, the postoperative maximum AST values were higher in the 2nd Era group than in the 1st Era group (median: 393 vs. 331 U/L, p < 0.05). However, the VAS scores at rest on postoperative day 1 and during movement on postoperative days 1, 2, and 3 were significantly lower in the 2nd Era group than in the 1st Era group (median: 22.5 vs. 20, p < 0.05; median: 61 vs. 50, p < 0.01; median: 56 vs. 48, p < 0.05; and median: 51 vs. 45, p < 0.01, respectively).

**Table 2:** Surgical and postoperative outcomes in the 1st Era and 2nd Era groups.

Variables	Group 1st Era (n = 157)	Group 2nd Era (n = 217)	P-Value
Operative time (min)^a^	329 (166–695)	342 (128–919)	0.12
Blood loss (ml)^a^	400 (20–7798)	442 (10–4976)	0.22
Postoperative hospital days (day)^a^	12 (3–206)	12 (4–194)	0.32
Max AST (U/L)	331 (120–2042)	393 (69–3519)	<0.05
Clavien-Dindo grade ≥ III; Yes/No	20 (12%)/137	37 (17%)/180	0.25
Respiratory complication; Yes/No	17 (10%)/140	29 (13%)/188	0.46
PHLF; Yes/No	58 (36%)/99	69 (31%)/148	0.29
Perioperative mortality; Yes/No	1 (0.006%)/156	5 (0.02%)/212	0.20
VAS score POD1 (at rest)^a^	22.5 (0–100)	20 (0–100)	<0.05
VAS score POD2 (at rest)^a^	20 (0–100)	14 (0–100)	0.05
VAS score POD3 (at rest)^a^	15 (0–83)	10 (0–82)	0.28
VAS score POD1 (during movement)^a^	61 (0–100)	50 (0–100)	<0.01
VAS score POD2 (during movement)^a^	56 (0–100)	48 (0–100)	<0.05
VAS score POD3 (during movement)^a^	51 (0–100)	45 (0–100)	<0.01

The 1st Era group (L-shaped incision: n = 123; Midline incision: n = 7; Laparoscopic surgery: n = 27) comprised patients who underwent procedures prior to the institutional policy change. The 2nd Era group (L-shaped incision: n = 66; Midline incision: n = 109; Laparoscopic surgery: n = 42) comprised patients who underwent procedures after the policy change.

AST, aspartate aminotransferase; PHLF, post-hepatectomy liver failure; VAS, visual analog scale; POD, postoperative day.

a [median, (range)].

### 3.3 Between-group propensity-score matched 1st L-shaped group and 2nd midline incision group comparisons

The 1st L-shaped group (1st L group) included those who may have been eligible to undergo a midline incision but underwent procedures that used L-shaped incisions. The 2nd midline group (2nd M group) included those who underwent procedures utilizing a midline incision. Patient characteristics are summarized in Supplemental Table 2. Clinical characteristics showed that the 1st L group had a lower prothrombin time (PT) (median (%): 83 vs. 90, p < 0.01), larger tumor size (median: 25 vs. 24 mm, p < 0.05), and heavier resection weight (median: 184 vs. 137 g, p < 0.05) than did the 2 nd M group. Surgical and postoperative outcomes are shown in Supplemental Table 3. The VAS scores on postoperative days 1 and 2 at rest and postoperative days 1, 2, and 3 during movement were significantly lower in the 2 nd M group than in the 1st L group (median: 22 vs. 18, p < 0.05; median: 20 vs. 11, p < 0.05; median: 60 vs. 48, p < 0.05; median: 55 vs. 45, p < 0.01; and median: 52 vs. 40, p < 0.05, respectively). However, there may be a case selection bias between the two groups due to historical background since differences in tumor size, resected weight, and liver function were observed between the two groups. One-to-one matching with propensity scores was used to overcome these effects. The characteristics of the matched patient groups are summarized in Tables 3 and 75 patients from each group were matched following covariate adjustment with the surgical and postoperative outcomes shown in Table 4. The results of the matched-group analysis were similar to those of the previous ITT analysis. The VAS scores at postoperative days 1 and 2 at rest and postoperative days 1, 2, and 3 during movement were significantly lower in the 2 nd M group than in the 1st L group (median: 22 vs. 15, p < 0.05; median: 20 vs. 10.5, p < 0.05; median: 53 vs. 41, p < 0.05; median: 56 vs. 44.5, p < 0.01; and median: 50 vs. 40, p < 0.05, respectively).

**Table 3:** Clinical characteristics of patients in the 1st L-shaped and 2nd Midline groups after propensity score matching.

Variables	Group 1st L-shaped (n = 75)	Group 2nd Midline (n = 75)	P-Value
Age^a^	70 (45–88)	71 (38–88)	0.85
Sex; Male/Female	59/16	50/25	0.09
BMI^a^	22.8 (16.9–37.3)	22.6 (16.1–31.8)	0.70
DM; Yes/No	25/29	22/37	0.33
T-Bil (mg/dl)^a^	0.8 (0.3–1.9)	0.8 (0.3–2.7)	0.99
AST (IU/l)^a^	37 (12–151)	31 (12–91)	0.08
Albumin (mg/dl)^a^	4.0 (2.5–5.1)	4.0 (2.7–4.9)	0.77
Prothrombin time (%)^a^	85 (66–112)	89 (49–119)	0.86
ICGR-15 (%)^a^	14.0 (3.5–79.1)	13.5 (3.1–54.6)	0.61
Child-Pugh classification; A/B	73/2	71/4	0.40
AFP (ng/ml)^a^	12.7 (0.5–2226)	7.1 (0.5–23800)	0.20
Tumor size (mm)^a^	25 (8–170)	25 (10–160)	0.53
Tumor number; Single/Multiple	48/27	47/28	0.86
Liver cirrhosis; Yes/No	17/58	18/57	0.84
Posterior region; Yes/No	35/40	39/36	0.51
Resected weight (g)^a^	156 (12–2332)	136 (6–974)	0.44
Type of hepatectomy; Anatomical/Limited	59/16	56/19	0.56
Type of hepatectomy; Major/Minor	3/72	7/68	0.19
Perioperative pain management; Epi-PCA/iv-PCA	42/33	39/36	0.62

The 1st L-shaped group (1st L group) included those who may have been eligible to undergo a midline incision but underwent procedures that used L-shaped incisions. The 2nd midline group (2nd M group) included those who underwent procedures utilizing a midline incision.

BMI, body mass index; DM, diabetes mellitus; T-Bil, total bilirubin; AST, aspartate aminotransferase; ALT, alanine transaminase; ICGR-15, indocyanine green retention rate at 15 min; AFP, α-fetoprotein; Epi-PCA, epidural–patient-controlled analgesia; iv-PCA, intravenous–patient-controlled analgesia.

a [median, (range)].

**Table 4:** Surgical and postoperative outcomes in the 1st L-shaped and 2nd Midline groups after propensity score matching.

Variables	Group 1st L-shaped (n = 75)	Group 2nd Midline (n = 75)	P-Value
Operative time (min)^a^	323 (166–537)	330 (128–919)	0.34
Blood loss (ml)^a^	469 (50–4470)	448 (20–4045)	0.66
Postoperative hospital days (day)^a^	12 (3–206)	12 (8–93)	0.58
Max AST (U/L)	336 (121–2042)	379 (69–2667)	0.85
Clavien-Dindo grade ≥ III; Yes/No	9 (12%)/66	13 (17%)/62	0.35
Respiratory complication; Yes/No	6 (8%)/69	10 (13%)/65	0.29
PHLF; Yes/No	30 (40%)/45	25 (33%)/50	0.39
VAS score POD1 (at rest)^a^	22 (0–100)	15 (0–86)	<0.05
VAS score POD2 (at rest)^a^	20 (0–80)	10.5 (0–80)	<0.05
VAS score POD3 (at rest)^a^	12 (0–83)	7 (0–60)	0.24
VAS score POD1 (during movement)^a^	53 (0–100)	41 (0–100)	<0.01
VAS score POD2 (during movement)^a^	56 (0–100)	44.5 (0–100)	<0.05
VAS score POD3 (during movement)^a^	50 (0–95)	40 (0–100)	<0.01

The 1st L-shaped group (1st L group) included those who may have been eligible to undergo a midline incision but underwent procedures that used L-shaped incisions. The 2nd midline group (2nd M group) included those who underwent procedures utilizing a midline incision.

AST, aspartate aminotransferase; PHLF, post-hepatectomy liver failure; VAS, visual analog scale; POD, postoperative day.

a [median, (range)].

### 3.4 Comparisons between midline incisions and laparoscopic surgery

The institutional change in treatment policy was made to minimize the invasiveness of surgeries. We examined the overall invasiveness of midline incisions by comparing midline incision surgeries to laparoscopic surgeries in the 2nd Era group, and patient characteristics are summarized in Supplemental Table 4. The laparoscopic surgery group had a smaller tumor size, lighter resection weight, and higher limited resection rate than did the midline incision group. Supplemental Table 5 shows the surgical and postoperative outcomes. Laparoscopic surgery groups had significantly less bleeding and shorter postoperative hospital stays. There were no significant differences between the two groups for postoperative complications. However, the VAS scores on postoperative day 1 at rest and postoperative day 1 during movement were lower in the midline incision group than in the laparoscopic surgery group (median: 18 vs. 30, p < 0.05 and median: 48 vs. 58, p < 0.01, respectively). This would suggest that the midline incision group had comparable invasiveness to the laparoscopic surgery group.

### 3.5 Risk factors for transition to L-shaped incisions

Finally, we examined the risk of transition to an L-shaped incision by comparing the midline and L-shaped incision groups in the 2nd Era group. Univariate analysis using preoperatively evaluated factors found that significant risk factors included male sex (p < 0.01), BMI ≥23 (p < 0.05), tumor size ≥30 mm (p < 0.01), multiple tumors (p < 0.05), hepatitis C virus (HCV) negative status (p < 0.01), liver damage B (p < 0.05), and major hepatectomy (p < 0.05, Table 5). In addition, multivariate analysis using these factors (Table 5) found that independent risk factors included male sex (p < 0.05), tumor size ≥30 mm (p < 0.01), and liver damage B (p < 0.05).

**Table 5:** Risk factors for transition from midline to L-shaped incisions according to univariate and multivariate analyses.

		Univariate analysis			Multivariate analysis		
Factors	Category	2nd Era Midline	2nd Era L-shaped	P-Value	Odds Ratio	95% CI	P-Value
Age	<75	71 (66%)	47 (71%)	0.40			
	≥75	38 (34%)	19 (29%)				
Sex	Male	76 (70%)	60 (90%)	<0.01	3.36	1.22–9.26	<0.05
	Female	33 (30%)	6 (10%)				
BMI	<23	55 (50%)	22 (33%)	<0.05	1.87	0.91–3.82	0.08
	≥23	54 (50%)	44 (67%)				
Tumor size	<30 mm	73 (67%)	20 (30%)	<0.01	4.5	2.11–9.58	<0.01
	≥30 mm	36 (33%)	46 (70%)				
Tumor number	Single	71 (65%)	33 (50%)	<0.05	1.51	0.74–3.07	0.25
	Multiple	38 (35%)	33 (50%)				
Posterior resection	Yes	53 (49%)	40(61%)	0.12			
	No	56 (51%)	26 (39%)				
Excision range	Anatomical	86 (79%)	57 (86%)	0.20			
	Limited	23 (21%)	9 (14%)				
Liver Cirrhosis	Present	22 (20%)	12 (18%)	0.74			
	Absent	87 (80%)	54 (82%)				
HBV	Positive	15 (14%)	6 (10%)	0.35			
	Negative	94 (86%)	60 (90%)				
HCV	Positive	59 (54%)	21 (32%)	<0.01	1.95	0.92–4.10	0.07
	Negative	50 (46%)	45 (68%)				
Liver damage	A	89 (82%)	45 (68%)	<0.05	2.43	1.05–5.58	<0.05
	B	20 (18%)	21 (32%)				
Type of Hx	Major	9 (8%)	13 (20%)	<0.05	1.26	0.44–3.59	0.65
	Minor	100 (92%)	53 (80%)				

*BMI, Body mass index; HBV, hepatitis B Virus; HCV, hepatitis C virus; CI, confidence interval.

## 4 Discussion

This study is the first to examine the outcomes associated with midline hepatectomy for right-sided liver cancers. The inverted L-shaped incision is currently the most common approach used for hepatectomy in cases of liver cancer. This approach is advantageous as it provides a visual field that readily allows right lobe mobilization, deep segment resection, and dissection of the short hepatic vein. While the inverted L-shaped incision is relatively less invasive than the reverse T-shaped incision, procedures utilizing L-shaped incisions tend to require increased analgesia, longer postoperative hospitalization time, and poor cosmetic outcomes resulting from scarring [30,31]. When compared to open surgeries, laparoscopic surgeries do not negatively affect surgical outcomes or cancer prognosis [[1], [2], [3], [4], [5], [6]]. The reduced invasiveness of laparoscopic hepatectomies is a major advantage of this procedure, and laparotomies are performed in cases with cirrhosis or highly advanced liver cancer or in those requiring a complicated systematic resection. Several reports have described the utility of hepatectomy versus upper midline incisions. However, these mainly focused on hepatectomies performed for harvesting living donor grafts, and very few report the efficacy of these procedures in liver cancer cases [30,31]. Hepatectomies performed using upper midline incisions are difficult in patients with liver cancer, particularly in cases requiring right-sided hepatectomy and anatomical resection primarily due to a limited surgical field of view and associated difficulties in identifying the excision range. Hepatectomy procedures utilizing the upper midline incision require the liver to be sufficiently dissected from its neighboring structures. Once adequate exposure is achieved, this procedure becomes similar to a conventional hepatectomy. The Thompson retractor and sufficient mobilization of left lobe volume, in small increments with IVC as the axis, improve the surgical field of view. Additionally, detachment of the left lobe promotes liver mobilization, allowing the organ to rotate around the IVC.

We compared the 1st and 2nd Era groups using an ITT protocol, changing only the surgical procedure selection policies without modifying surgery indications. We found that overall pain scores improved following the implementation of the policy changes. This improvement may have been due to the fact that hepatectomy using a midline incision avoids cutting of the rectus abdominis muscle; therefore, an increase in midline incision utilization would reduce the overall pain associated with rectus abdominis manipulation. However, high postoperative AST levels and potential brute force associated with midline incision use may be the disadvantages of this policy change.

Comparisons between the 1st L and 2 nd M groups allowed for clarification of advantages and disadvantages associated with the use of a midline incision, thereby allowing us to distinguish the effects of the new treatment policy since there were no differences in postoperative results between the two groups, even after propensity score matching. We also found that postoperative pain associated with midline incision procedures was comparatively lower, and differences in postoperative maximum AST levels were not observed in this analysis, contrary to the ITT analysis results. Therefore, this analysis did not allow us to determine whether liver damage was associated with liver excision in right-sided hepatectomy procedures utilizing a small midline incision.

Laparoscopic surgery was found to be advantageous with regard to hospital stay period and decreased bleeding. Further, laparoscopic surgery cases tended to recover faster. Therefore, it is advantageous to continue performing laparoscopic surgery in eligible cases. Surprisingly, using midline incision and laparoscopic surgery was comparable with regard to pain-associated outcomes. However, these results are limited because they were obtained through sub-analyses and background factors for both groups were not complete. Therefore, we cannot conclude that midline incisions are less painful than laparoscopic hepatectomy. Nonetheless, these results indicate that midline incisions do not significantly increase postoperative pain compared to laparoscopic hepatectomy.

Risk factors associated with the intraoperative conversion of midline incisions to L-shaped incisions included male sex, tumor diameter, and overall liver damage status, likely because compared to women, men have a relatively deeper abdominal cavity, which would, in some cases, necessitate the use of an L-shaped incision to secure a visual field. Even in cases with a larger tumor diameter, L-shaped incisions have a higher probability of securing an adequate visual field. Additionally, cases with extensive liver damage may require L-shaped incisions for controlling excessive bleeding.

In summary, we found that the use of hepatectomy through an upper midline incision was technically feasible and safe in patients with liver cancer, providing relatively decreased postoperative pain along with similar outcomes to hepatectomy cases utilizing conventional inverted L-shaped incisions. In addition, comparisons between midline incisions and laparoscopic surgeries found that while laparoscopic procedures were associated with decreased bleeding and length of hospital stays, midline incision procedures were associated with relatively lower postoperative pain levels.

In cases where laparotomy is required for hepatectomy, midline incision has considerable advantages in terms of pain. Therefore, in open surgery, it appears better to start the surgery with a midline incision. However, this study found that in men, tumor diameter and liver damage are risk factors of midline incision difficulty. Therefore, even if surgery is started with a midline incision, it may be desirable to move to an L-shaped incision carefully when there is such a risk factor.

In conclusion, this retrospective study demonstrates that midline incision hepatectomies, while not as efficacious as laparoscopic procedures, are feasible for cases of right-sided hepatectomy and may provide benefits compared to procedures utilizing L-shaped incisions.

## Provenance and peer review

Not commissioned, externally peer-reviewed.

## Disclosure of commercial interest

None of the authors has any commercial interests associated with this study or received any financial or material support for this study.

## Funding

All the authors declare that they have no conflict of interest.

## Sources of funding

This research did not receive any specific grant from funding agencies in the public, commercial, or not-for-profit sectors.

## Ethical approval

This study conforms to the provisions of the Declaration of Helsinki and was approved by the local Institutional Review Board (E-1580). All patients provided informed consent prior to undergoing their respective surgical procedures.

## Research registration unique identifying number (UIN)

Name of the registry: Research Registry

Unique Identifying number or registration ID: researchregistry6476

Hyperlink to your specific registration (must be publicly accessible and will be checked): https://www.researchregistry.com/register-now#user-researchregistry/registerresearchdetails/6007c7d7dbe65d001cb358ac/

## Author contribution

Conceptualization; Daisuke Takei, Shintaro Kuroda, Keiso Matsubara, Hiroaki Mashima, Masakazu Hashimoto, Tsuyoshi Kobayashi, Hideki Ohdan

Data curation; Daisuke Takei, Keiso Matsubara, Hiroaki Mashima

Formal analysis; Daisuke Takei

Investigation; Daisuke Takei, Shintaro Kuroda

Methodology; Daisuke Takei, Shintaro Kuroda, Tsuyoshi Kobayashi, Hideki Ohdan

Project administration; Hideki Ohdan

Supervision; Shintaro Kuroda

Validation; Daisuke Takei, Shintaro Kuroda, Tsuyoshi Kobayashi, Hideki Ohdan

Roles/Writing - original draft; Daisuke Takei

Writing - review & editing; Shintaro Kuroda, Tsuyoshi Kobayashi, Hideki Ohdan

## Guarantor

Daisuke Takei, Shintaro Kuroda, Keiso Matsubara, Hiroaki Mashima, Masakazu Hashimoto, Tsuyoshi Kobayashi, Hideki Ohdan

## Declaration of competing interest

This research did not receive any specific grant from funding agencies in the public, commercial, or not-for-profit sectors.

## References

[1] Nelson H., Sarget D.J., Wieand H.S., Fleshman J., Anvari M., Stryker S.J., Beart R.W., Hellinger M., Flanagan R., Peters W., Ota D., Clinical Outcomes of Surgical Therapy Study Group (2004). A comparison of laparoscopically assisted and open colectomy for colon cancer. N. Engl. J. Med..

[2] Fleshman J., Sargent D.J., Green E., Anvari M., Stryker S.J., Beart R.W., Hellinger M., Flanagan R., Peters W., Nelson H. (2007). Clinical Outcomes of Surgical Therapy Study Group, Laparoscopic colectomy for cancer is not inferior to open surgery based on 5-year data from the COST Study Group trial. Ann. Surg..

[3] Hu Y., Huang C., Sun Y., Su X., Cao H., Hu J., Xue Y., Suo J., Tao K., He X., Wei H., Ying M., Hu W., Du X., Chen P., Liu H., Zheng C., Liu F., Yu J., Li Z., Zhao G., Chen X., Wang K., Li P., Xing J., Li G. (2016). Morbidity and mortality of laparoscopic versus open D2 distal gastrectomy for advanced gastric cancer: a randomized controlled trial. J. Clin. Oncol..

[4] Kaiser A.M., Kang J.C., Chan L.S., Vukasin P., Beart R.W. (2004). Laparoscopic-assisted vs. open colectomy for colon cancer: a prospective randomized trial. J. Laparoendosc. Adv. Surg. Tech. A..

[5] Lacy A.M., Garcia-Valdecasas J.C., Delgado S., Castells A., Taura P., Pique J.M., Visa J. (2002). Laparoscopy-assisted colectomy versus open colectomy for treatment of non-metastatic colon cancer: a randomised trial. Lancet.

[6] Leung K.L., Kwok S.P., Lam S.C., Lee J.F., Yiu R.Y., Ng S.S., Lai P.B., Lau W.Y. (2004). Laparoscopic resection of rectosigmoid carcinoma: prospective randomised trial. Lancet.

[7] Abu Hilal M., Underwood T., Taylor M.G., Hamdan K., Elberm H., Pearce N.W. (2010). Bleeding and hemostasis in laparoscopic liver surgery. Surg. Endosc..

[8] Ban D., Tanabe M., Ito H., Otsuka Y., Nitta H., Abe Y., Hasegawa Y., Katagiri T., Takagi C., Itano O., Kaneko H., Wakabayashi G. (2014). A novel difficulty scoring system for laparoscopic liver resection. J. Hepatobiliary Pancreat. Sci..

[9] Buell J.F., Thomas M.T., Rudich S., Marvin M., Nagubandi R., Kadiyala V., Brock G., McMasters K.M. (2008). Experience with more than 500 minimally invasive hepatic procedures. Ann. Surg..

[10] Cho J.Y., Han H.S., Yoon Y.S., Shin S.H. (2008). Feasibility of laparoscopic liver resection for tumors located in the posterosuperior segments of the liver, with a special reference to overcoming current limitations on tumor location. Surgery.

[11] Kasai M., Cipriani F., Gayet B., Aldrighetti L., Ratti F., Sarmiento J.M., Scatton O., Kim K.H., Dagher I., Topal B., Primrose J., Nomi T., Fuks D., Abu Hilal M. (2018). Laparoscopic versus open major hepatectomy: a systematic review and meta-analysis of individual patient data. Surgery.

[12] Morino M., Morra I., Miglietta C., Garrone C. (2003). Laparoscopic vs open hepatic resection: a comparative study. Surg. Endosc..

[13] Ziogas I.A., Tsoulfas G. (2017). Advances and challenges in laparoscopic surgery in the management of hepatocellular carcinoma. World J. Gastrointest. Surg..

[14] D. Ban, M. Tanabe, H. Kumamaru, H. Nitta, Y. Otsuka, H. Miyata, Y. Kakeji, Y. Kitagawa, H. Kaneko, G. Wakabayashi, H. Yamaue, M. Yamamoto, Safe dissemination of laparoscopic liver resection in 27,146 cases between 2011 and 2017 from the National Clinical Database of Japan, Ann. Surg. 2020 Mar 20. 10.1097/SLA.0000000000003799. Online ahead of print. 32209896

[15] Demirbas T., Bulutcu F., Dayangac M., Yaprak O., Guler N., Oklu L., Akyildiz M., Altaca G., Tokat Y., Yuzer Y. (2013). Which incision is better for living-donor right hepatectomy? Midline, J-shaped, or Mercedes. Transplant. Proc..

[16] Ikegami T., Harimoto N., Shimokawa M., Yoshizumi T., Uchiyama H., Itoh S., Okabe N., Sakata K., Nagatsu A., Soejima Y., Maehara Y. (2016). The learning curves in living donor hemiliver graft procurement using small upper midline incision. Clin. Transplant..

[17] Kim S.H., Kim Y.K. (2012). Living donor right hepatectomy using the hanging maneuver by Glisson's approach under the upper midline incision. World J. Surg..

[18] Makki K., Chorasiya V.K., Sood G., Srivastava P.K., Dargan P., Vij V. (2014). Laparoscopy-assisted hepatectomy versus conventional (open) hepatectomy for living donors: when you know better, you do better. Liver Transplant..

[19] Nagai S., Brown L., Yoshida A., Kim D., Kazimi M., Abouljoud M.S. (2012). Mini-incision right hepatic lobectomy with or without laparoscopic assistance for living donor hepatectomy. Liver Transplant..

[20] Shirabe K., Eguchi S., Okajimi H., Hasegawa K., Marubashi S., Umeshita K., Kawasaki S., Yanaga K., Shimada M., Kaido T., Kawagishi N., Taketomi A., Mizuta K., Kokudo N., Uemoto S., Maehara Y. (2018). Japanese Liver Transplantation Society, Current status of surgical incisions used in donors during living related liver transplantation-a nationwide survey in Japan. Transplantation.

[21] Soyama A., Takatsuki M., Hidaka M., Adachi T., Kitasato A., Kinoshita A., Natsuda K., Baimakhanov Z., Kuroki T., Eguchi S. (2015). Hybrid procedure in living donor liver transplantation. Transplant. Proc..

[22] Suh S.W., Lee K.W., Lee J.M., Choi Y.R., Yi N.J., Suh K.S. (2015). Clinical outcomes of and patient satisfaction with different incision methods for donor hepatectomy in living donor liver transplantation. Liver Transplant..

[23] Takasaki K. (1998). Glissonean pedicle transection method for hepatic resection: a new concept of liver segmentation. J. Hepatobiliary Pancreat. Surg..

[24] Takayama T., Makuuchi M. (1996). Intraoperative ultrasonography and other techniques for segmental resections. Surg. Oncol. Clin..

[25] Keele K.D. (1948). The pain chart. Lancet.

[26] Nanashima A., Sumida Y., Morino S., Yamaguchi H., Tanaka K., Shibasaki S., Ide N., Sawai T., Yasutake T., Nakagoe T., Nagayasu T. (2004). The Japanese integrated staging score using liver damage grade for hepatocellular carcinoma in patients after hepatectomy. Eur. J. Surg. Oncol..

[27] Dindo D., Demartines N., Clavien P.A. (2004). Classification of surgical complications: a new proposal with evaluation in a cohort of 6336 patients and results of a survey. Ann. Surg..

[28] Rahbari N.N., Garden O.J., Padbury R., Brooke-Smith M., Crawford M., Adam R., Koch M., Makuuchi M., Dematteo R.P., Christophi C., Banting S., Usatoff V., Nagino M., Maddern G., Hugh T.J., N Vauthey J., Greig P., Rees M., Yokoyama Y., Fan S.T., Nimura Y., Figueras J., Capussotti L., Büchler M.W., Weitz J. (2011). Posthepatectomy liver failure: a definition and grading by the international study group of liver surgery (ISGLS). Surgery.

[29] Agha R., Abdall-Razak A., Crossley E., Dowlut N., Iosifidis C., Mathew G. (2019). For the STROCSS group, the STROCSS 2019 guideline: strengthening the reporting of cohort studies in surgery. Int. J. Surg..

[30] Kim S.H., Kim Y.K. (2013). Upper midline incision for liver resection. HPB.

[31] Soyama A., Takatsuki M., Adachi T., Kitasato A., Torashima Y., Natsuda K., Tanaka T., Yamaguchi I., Tanaka S., Kinoshita A., Kuroki T., Eguchi S. (2014). A hybrid method of laparoscopic-assisted open liver resection through a short upper midline laparotomy can be applied for all types of hepatectomies. Surg. Endosc..

