# Supplementary material for: Usefulness and safety of midline incision for right-sided hepatectomy: Cohort study
Source: Ann Med Surg (Lond). 2021 Jun 13;67:102498. doi: 10.1016/j.amsu.2021.102498 (PMC8246149; doi:10.1016/j.amsu.2021.102498)
Supplement: Multimedia component 1 [file mmc1.docx]

**Supplemental Table 1.** Clinical characteristics of the entire population

| **Variables** | **N = 374** |
| --- | --- |
| Age * | 70 (20-91) |
| Sex; Male/Female | 287/87 |
| BMI * | 23.1 (15.5-37.3) |
| DM; Yes/No | 114/181 |
| Background liver; hepatitis B/hepatitis C/other | 57/175/142 |
| T-Bil (mg/dl) * | 0.8 (0.3-2.9) |
| AST (IU/l) * | 33 (11-390) |
| Albumin (mg/dl) * | 4.0 (2.3-7.4) |
| Prothrombin time (%) * | 86.5 (24-145) |
| ICGR 15 (%) * | 13.6 (2.2-79.2) |
| Child-Pugh classification; A/B | 348/26 |
| AFP (ng/ml) * | 9.0 (0.5-290700) |
| Tumor size (mm) * | 25 (8-200) |
| Tumor number; Single/Multiple | 230/144 |
| Liver cirrhosis; Yes/No | 75/299 |
| Posterior region; Yes/No | 190/184 |
| Resected weight (g) * | 140 (5-2903) |
| Type of hepatectomy; Anatomical/Limited | 261/113 |
| Type of hepatectomy; Major/Minor | 38/336 |
| Perioperative pain management; Epi-PCA/iv-PCA | 193/180 |
| Perioperative mortality; Yes/No | 5/369 |

* [median, (range)]

BMI, body mass index; DM, diabetes mellitus; T-Bil, total bilirubin; AST, aspartate aminotransferase; ALT, alanine transaminase; ICGR 15, indocyanine green retention rate at 15 minutes; AFP, α-fetoprotein; Epi-PCA, epidural–patient-controlled analgesia; iv-PCA, intravenous–patient-controlled analgesia

| **Variables** | **Group 1^st^**  **L-shaped**  **(n=123)** | **Group 2^nd^ Midline**  **(n=109)** | **P-Value** |
| --- | --- | --- | --- |
| Age * | 70 (33-88) | 71 (38-88) | 0.35 |
| Sex; Male/Female | 97/26 | 76/33 | 0.11 |
| BMI * | 22.7 (16.5-37.3) | 22.9 (15.7-31.8) | 0.81 |
| DM; Yes/No | 38/54 | 32/57 | 0.46 |
| T-Bil (mg/dl) * | 0.8 (0.3-2.6) | 0.8 (0.3-2.7) | 0.88 |
| AST (IU/l) * | 36 (12-296) | 31 (12-104) | 0.06 |
| Albumin (mg/dl) * | 4.1 (2.3-5.1) | 4.0 (2.7-5.2) | 0.92 |
| Prothrombin time (%) * | 83 (24-112) | 90 (24-126) | <0.01 |
| ICGR-15 (%) * | 13.8 (3.5-79.1) | 13.5 (2.2-79.2) | 0.63 |
| Child-Pugh classification; A/B | 115/8 | 104/5 | 0.40 |
| AFP (ng/ml) * | 13.5 (0.5-11234) | 7.1 (0.5-23800) | 0.05 |
| Tumor size (mm) * | 25 (8-170) | 24 (10-160) | <0.05 |
| Tumor number; Single/Multiple | 71/52 | 71/38 | 0.24 |
| Liver cirrhosis; Yes/No | 26/97 | 22/87 | 0.85 |
| Posterior region; Yes/No | 54/69 | 53/56 | 0.47 |
| Resected weight (g) * | 184 (11-2332) | 137 (6-974) | <0.05 |
| Type of hepatectomy; Anatomical/Limited | 96/27 | 86/23 | 0.87 |
| Type of hepatectomy; Major/Minor | 16/107 | 9/100 | 0.24 |
| Perioperative pain management; Epi-PCA/iv-PCA | 63/60 | 60/49 | 0.56 |

**Supplemental Table 2. Clinical characteristics of patients in the 1^st^ L-shaped and 2^nd^ Midline groups**

The 1st L-shaped group (1st L group) included those who may have been eligible to undergo a midline incision but underwent procedures that used L-shaped incisions. The 2nd midline group (2^nd^ M group) included those who underwent procedures utilizing a midline incision.

* [median, (range)]

BMI, body mass index; DM, diabetes mellitus; T-Bil, total bilirubin; AST, aspartate aminotransferase; ALT, alanine transaminase; ICGR-15, indocyanine green retention rate at 15 minutes; AFP, α-fetoprotein; Epi-PCA, epidural–patient-controlled analgesia; iv-PCA, intravenous–patient-controlled analgesia

**Supplemental Table 3. Surgical and postoperative outcomes in the 1^st^ L-shaped and 2^nd^ Midline groups**

| **Variables** | **Group 1^st^**  **L-shaped**  **(n=123)** | **Group 2^nd^ Midline**  **(n=109)** | **P-Value** |
| --- | --- | --- | --- |
| Operative time (min) * | 332 (166-695) | 306 (128-919) | 0.41 |
| Blood loss (ml) * | 430 (40-4470) | 457 (20-4045) | 0.84 |
| Postoperative hospital days (day) * | 13 (3-206) | 12 (8-93) | 0.83 |
| Max AST (U/L) | 378 (120-2042) | 379 (69-3105) | 0.70 |
| Clavien-Dindo grade ≥ III; Yes/No | 18 (14%)/105 | 18 (16%)/91 | 0.69 |
| Respiratory complication; Yes/No | 14 (11%)/109 | 13 (11%)/96 | 0.89 |
| PHLF; Yes/No | 47 (38%)/76 | 32 (29%)/77 | 0.15 |
| VAS score POD1 (at rest) * | 22 (0-100) | 18 (0-86) | <0.05 |
| VAS score POD2 (at rest) * | 20 (0-80) | 11 (0-80) | <0.05 |
| VAS score POD3 (at rest) * | 15 (0-83) | 10 (0-82) | 0.06 |
| VAS score POD1 (during movement) * | 60 (0-100) | 48 (0-100) | <0.01 |
| VAS score POD2 (during movement) * | 55 (0-100) | 45 (0-100) | <0.05 |
| VAS score POD3 (during movement) * | 52 (0-95) | 40 (0-100) | <0.01 |

The 1st L-shaped group (1st L group) included those who may have been eligible to undergo a midline incision but underwent procedures that used L-shaped incisions. The 2nd midline group (2^nd^ M group) included those who underwent procedures utilizing a midline incision.

* [median, (range)]

AST, aspartate aminotransferase; PHLF, post-hepatectomy liver failure; VAS, visual analog scale; POD, postoperative day

**Supplemental Table 4. Clinical characteristics of patients in the 2^nd^ Midline and 2^nd^ Lapa groups**

| **Variables** | **Group 2^nd^ Midline**  **(n=109)** | **Group 2^nd^ Lapa**  **(n=42)** | **P-Value** |
| --- | --- | --- | --- |
| Age * | 71 (38-88) | 71.5 (20-86) | 0.76 |
| Sex; Male/Female | 76/33 | 29/13 | 0.93 |
| BMI * | 22.9 (15.7-31.8) | 23.2 (17.8-36.5) | 0.30 |
| DM; Yes/No | 32/57 | 13/26 | 0.77 |
| T-Bil (mg/dl) * | 0.8 (0.3-2.7) | 0.85 (0.4-2.6) | 0.59 |
| AST (IU/l) * | 31 (12-104) | 26.5 (14-61) | <0.05 |
| Albumin (mg/dl) * | 4.0 (2.7-5.2) | 4.35 (2.5-7.4) | 0.10 |
| Prothrombin time (%) * | 90 (24-126) | 89.5 (61-145) | 0.42 |
| ICGR 15 (%) * | 13.5 (2.2-79.2) | 12.4 (4.8-44.6) | 0.31 |
| Child-Pugh classification; A/B | 104/5 | 40/2 | 0.96 |
| AFP (ng/ml) * | 7.1 (0.5-23800) | 3.5 (1-60.4) | <0.01 |
| Tumor size (mm) * | 24 (10-160) | 17.5 (8-50) | <0.01 |
| Tumor number; Single/Multiple | 71/38 | 29/13 | 0.64 |
| Liver cirrhosis; Yes/No | 22/87 | 5/37 | 0.23 |
| Posterior region; Yes/No | 56/53 | 22/20 | 0.91 |
| Resected weight (g) * | 137 (6-974) | 44.5 (8-251) | <0.01 |
| Type of hepatectomy; Anatomical/Limited | 86/23 | 11/31 | <0.01 |
| Type of hepatectomy; Major/Minor | 9/100 | 0/42 | 0.05 |
| Perioperative pain management; Epi-PCA/iv-PCA | 60/49 | 21/21 | 0.57 |

The 2nd Midline group included those who underwent procedures utilizing a midline incision. The 2nd Lapa group included those who underwent a laparoscopic procedure.

* [median, (range)]

BMI, body mass index; DM, diabetes mellitus; T-Bil, total bilirubin; AST, aspartate aminotransferase; ALT, alanine transaminase; ICGR-15, indocyanine green retention rate at 15 minutes; AFP, α-fetoprotein; Epi-PCA, epidural–patient-controlled analgesia; iv-PCA, intravenous–patient-controlled analgesia

**Supplemental Table 5. Surgical and postoperative outcomes in the 2^nd^ Midline and 2^nd^ Lapa groups**

|  | **Group 2^nd^ Midline**  **(n=109)** | **Group 2^nd^ Lapa**  **(n=42)** | **P-Value** |
| --- | --- | --- | --- |
| Operative time (min) * | 306 (128-919) | 317 (149-682) | 0.86 |
| Blood loss (ml) * | 457 (20-4045) | 102 (10-2750) | <0.01 |
| Postoperative hospital days (day) * | 12 (8-93) | 10 (4-45) | <0.01 |
| Max AST (U/L) | 379 (69-3105) | 373 (126-3519) | 0.97 |
| Clavien-Dindo grade ≥ III; Yes/No | 18 (16%)/91 | 3 (7%)/39 | 0.13 |
| Respiratory complication; Yes/No | 13 (11%)/96 | 2 (4%)/40 | 0.18 |
| PHLF; Yes/No | 32 (29%)/77 | 10 (23%)/32 | 0.49 |
| Perioperative mortality; Yes/No | 2 (1%)/107 | 1 (2%) /41 | 0.82 |
| VAS score POD1 (at rest) * | 18 (0-86) | 30 (0-100) | <0.05 |
| VAS score POD2 (at rest) * | 11 (0-80) | 20 (0-85) | 0.16 |
| VAS score POD3 (at rest) * | 10 (0-82) | 10 (0-80) | 0.54 |
| VAS score POD1 (with movement) * | 48 (0-100) | 58 (0-100) | <0.01 |
| VAS score POD2 (with movement) * | 45 (0-100) | 50 (10-100) | 0.20 |
| VAS score POD3 (with movement) * | 40 (0-100) | 45.5 (5-100) | 0.27 |

The 2nd Midline group included those who underwent procedures utilizing a midline incision. The 2nd Lapa group included those who underwent a laparoscopic procedure.

* [median, (range)]

AST, aspartate aminotransferase; PHLF, post-hepatectomy liver failure; VAS, visual analog scale; POD, postoperative day
